# Data Quality– and Utility-Compliant Anonymization of Common Data Model–Harmonized Electronic Health Record Data: Protocol for a Scoping Review

**DOI:** 10.2196/46471

**Published:** 2023-08-11

**Authors:** Gaetan Kamdje Wabo, Fabian Prasser, Kerstin Gierend, Fabian Siegel, Thomas Ganslandt

**Affiliations:** 1 Department of Biomedical Informatics Center for Preventive Medicine and Digital Health Baden-Württemberg Mannheim Medical Faculty of the University of Heidelberg Mannheim Germany; 2 Berlin Institute of Health at Charité Universitätsmedizin Berlin Berlin Germany; 3 Department of Urology and Urosurgery University Medical Center Mannheim Mannheim Medical Faculty of the University of Heidelberg Mannheim Germany; 4 Chair of Medical Informatics Friedrich-Alexander-Universität Erlangen-Nürnberg Erlangen Germany

**Keywords:** EHR, electronic health record, data quality, common data model, data standard, data privacy models, data anonymization

## Abstract

**Background:**

The anonymization of Common Data Model (CDM)–converted EHR data is essential to ensure the data privacy in the use of harmonized health care data. However, applying data anonymization techniques can significantly affect many properties of the resulting data sets and thus biases research results. Few studies have reviewed these applications with a reflection of approaches to manage data utility and quality concerns in the context of CDM-formatted health care data.

**Objective:**

Our intended scoping review aims to identify and describe (1) how formal anonymization methods are carried out with CDM-converted health care data, (2) how data quality and utility concerns are considered, and (3) how the various CDMs differ in terms of their suitability for recording anonymized data.

**Methods:**

The planned scoping review is based on the framework of Arksey and O'Malley. By using this, only articles published in English will be included. The retrieval of literature items should be based on a literature search string combining keywords related to data anonymization, CDM standards, and data quality assessment. The proposed literature search query should be validated by a librarian, accompanied by manual searches to include further informal sources. Eligible articles will first undergo a deduplication step, followed by the screening of titles. Second, a full-text reading will allow the 2 reviewers involved to reach the final decision about article selection, while a domain expert will support the resolution of citation selection conflicts. Additionally, key information will be extracted, categorized, summarized, and analyzed by using a proposed template into an iterative process. Tabular and graphical analyses should be addressed in alignment with the PRISMA-ScR (Preferred Reporting Items for Systematic Reviews and Meta-Analyses Extension for Scoping Reviews) checklist. We also performed some tentative searches on Web of Science for estimating the feasibility of reaching eligible articles.

**Results:**

Tentative searches on Web of Science resulted in 507 nonduplicated matches, suggesting the availability of (potential) relevant articles. Further analysis and selection steps will allow us to derive a final literature set. Furthermore, the completion of this scoping review study is expected by the end of the fourth quarter of 2023.

**Conclusions:**

Outlining the approaches of applying formal anonymization methods on CDM-formatted health care data while taking into account data quality and utility concerns should provide useful insights to understand the existing approaches and future research direction based on identified gaps. This protocol describes a schedule to perform a scoping review, which should support the conduction of follow-up investigations.

**International Registered Report Identifier (IRRID):**

PRR1-10.2196/46471

## Introduction

The anonymization of health data is a key approach for preserving patient anonymity during the secondary use of relational (ie, tabular) electronic health record (EHR) data [1]. However, to overcome the challenges related to the considerable heterogeneity in clinical data source systems (eg, due to diverse medical data coding frameworks, heterogeneous definitions of laboratory data values, or disparate setting- or task-dependent metadata), the use of common data models (CDMs) has been proposed and discussed [2]. Converting structured or unstructured source data to CDM standards helps to reach an understanding of commonly harmonized data into collaborative network research [3] and hence facilitates the cross-institutional exchange of medical data by using appropriate CDM metadata [2]. By approaching this, anonymization of CDM-converted EHR data promises patient privacy–secured sharing and analysis of harmonized data, which requires specific data anonymization components.

Extensive efforts describing the conduction [1,4-13] of data anonymization exist, and it is essential to differentiate and properly address 3 major aspects when dealing with relational data anonymization (anonymization of tabular data). This includes privacy models, data transformation models, and data utility models for assessing and ensuring the fitness of anonymous data for use. In terms of proposed privacy models, the k-anonymity privacy model [1,7] is one of the most widely used models. It consists of placing at least *k* patients in an equivalence class with the same patient-identifying data element values (so-called quasi-identifiers; eg, birthdate and zip code), so that the probability of reidentifying a patient becomes *1/k*. The value of the threshold *k* is determined by the data owner (eg, a hospital department sharing the data) depending on the size of the data and privacy protection level [1]. Because of the limitations of this model for fully protecting sensitive information (eg, patient health insurance and treating medical doctor), the *l*-diversity privacy model [1,8] was proposed. This ensures that at least *l*-“well-represented” values for sensitive data elements are presented within each equivalent class. Furthermore, additional data privacy models including the *t*-closeness privacy model [9] (for preventing linkage of the record and data elements) and the differential privacy model [10] (for preventing table linkage and probabilistic attacks) were also addressed. The strengths and limitations of these models were discussed in depth and extensively by Majeed and Lee [1] and Lei et al [11]. For implementing the data privacy models on data, a corresponding data transformation model is required, which may include a variety of technical operations. These comprise, for instance, generalization (by replacing some data values with parent values), suppression (implementing data record, value, or cell suppression), permutation (partitioning data records into dissociated groups), perturbation (partly or totally replacing original data with synthetic data), or anatomization (dissociating the relationships among patient-identifying data elements) [1,11,13]. Implementing the privacy- and data transformation models mentioned above leads to high impact on the quality of anonymous data in terms of utility. Nonetheless, utility models including metrics such as accuracy or error rate, the *F*-measure, precision, and recall have been proposed to assess the utility of anonymous data for special purposes [1]. Furthermore, the weighted certainty penalty, generalized information loss, the global loss penalty, relative error, or information theoretical metrics have also been recommended to estimate the utility of anonymous data for general purposes [1,12,13]. In addition, further evidence-based recommendations on how to assess and report on EHR data quality have been proposed [14-18] (eg, 3×3 data quality assessment guidelines [16], the framework of Kahn et al [15], or that of Fox et al [18]), and tools for data anonymization, transformation, and utility models have been proposed and discussed [4].

Among others, by using CDM standards in the clinical context, related source data can be more efficiently reused, organized, described, validated, searched, and queried [2]. International standards such as Fast Health Interoperability Resources (FHIR) [19] and CDM frameworks including the Informatics for Integrating Biology & the Bedside (i2b2) TranSMART CDM [20], the Observational Medical Outcomes Partnership’s Observational Health Data Sciences and Informatics (OMOP OHDSI) CDM [21], the Patient-Centered Outcomes Research network (PCORNet) CDM [22], and the Clinical Data Interchange Standards Consortium’s (CDISC’s) Operational Data Model (ODM) [23] therefore gained widespread attention in the scientific community in the last decades. For instance, the Medical Informatics in Research and Care in University Medicine (MIRACUM) consortium of the German Medical Informatics Initiative [24,25] presents an illustrative deployment of some of these CDMs.

While the interoperable conversion and querying of source EHR data into multiple CDM formats has been demonstrated [26], it is nonetheless worth noting that an entire transformation of health care data from the original data format to CDMs, or from one CDM to another one, is barely practicable [2]. This leads to potential challenges related to data completeness in the context of the use of CDM-converted health care data. Moreover, the relational anonymization of CDM-converted data by using the k-anonymity or *l*-diversity privacy models might build an interesting lever to allow patient privacy–preserved sharing of harmonized health care data as shown by Almeida et al [6] and in a recent study by Pitoglou et al [27]. Nonetheless, the anonymization of health care data can disproportionally affect the quality of resulting anonymous data sets due to information loss, and hence their suitability for medical research, as investigated by Langarizadeh et al [28] and Ferrão et al [29]. Especially in the case of CDM-converted data, anonymization may affect both cardinalities and completeness requirements of the respective CDM data models. This can be observed, for example, by the suppression of mandatory fields or by generalization through entering of ranges (eg, age range) into fields that only allow numeric values (not interval). Moreover, once CDM-converted data have been anonymized, it would be relevant to ensure whether the generated anonymous data may at all be stored in conformity with the CDM structures, or if it would be necessary to adapt the CDM specifications (eg, through some slicing in FHIR specifying both the exact and range-based anonymous age). This indicates the need for a thorough investigation of the suitability of CDM databases to record anonymized data in a quality-compliant format.

This raises problems related to how anonymization-assisted preservation of patient privacy in using or sharing of CDM-harmonized health care data with a reflection of anonymous data utility is addressed, and whether CDMs differ in terms of their ability to record anonymized data. Despite the large range of studies performed in the fields of relational data anonymization [1,4-13], CDM standards [19-22,30,31], and frameworks for medical data quality assessments [15-17,32], little attention has been paid to an extensive review of the existing literature addressing these questions. Reviewing the existing evidence concerning these issues might aid in identifying, describing, and understanding how relational data are anonymized, evaluated, and documented into specific CDM databases and to what extent the utility and quality of the obtained anonymous data are addressed. There could be some gaps in data utility research to be considered when anonymizing specific CDM-transformed clinical data for specific data mining scenarios such as predictive analysis or machine learning for improving health care quality. The evidence and identified gaps should serve as support for further investigations in the field of utility-compliant anonymizing of harmonized health care data.

Given this research scope, we plan to conduct a scoping review that aims to identify and describe (1) the current status and challenges of implementing formal privacy models (eg, k-anonymization, *l*-diversity, differential privacy, or *t*-closeness) on CDM databases (including i2b2, OMOP, CDISC, PCORnet, and FHIR), (2) the strategies used there to ensure the quality and utility of anonymized data, and (3) the differences in multiple CDM standards in relation to their suitability to record and document anonymized data.

## Methods

### Ethical Considerations

No ethics approvals are required since the planned study is only concerned with the assessment of the literature within a specific domain. Hence, no sensitive patient-identifying data will be processed.

### Schedule

For conducting this scoping review study, we will use the methodological framework of Arksey and O’Malley [33], which recommends an analysis process based on 5 steps: step 1—identifying the research question, step 2—identifying the relevant studies, step 3—selecting studies, step 4—extracting and charting data, and step 5—collating, summarizing, and reporting the results. Below, we describe the methodology’s stepwise concepts and the planned and already implemented in-between steps.

### Step 1—Identification of the Research Questions

As a prelude, an initial exploration of the literature was manually carried out to gain an overview of the issues regarding data quality and data anonymization as well as to determine the appropriate keywords to be included. A search was undertaken using a combination of the search terms “data quality,” “anonymi*ation,” and “deidentification,” and by querying the literature platforms PubMed and Web of Science Core Collection. The most relevant articles were selected and analyzed upon full-text reading. To form the final research questions, we additionally addressed an explicit focus on the most internationally adopted CDMs (including i2b2 TranSMART, OMOP OHDSI, PCORnet, and CDISC ODM) and the FHIR standard. The research questions were derived by considering both the research objectives stated above.

In doing so, the planned scoping review investigation will address the following 3 research questions: how are formal anonymization methods carried out with CDM-converted health care data and which challenges are observed? How are data quality and utility concerns considered during the anonymization of CDM-converted health care data? How does anonymization affect the specifications of different CDM data models, and which differences are observable in the CDMs regarding their suitability for recording and documenting anonymized data?

### Step 2—Identifying the Relevant Studies

#### Overview

To identify the most relevant articles matching the research questions, we will explore a large set of articles by taking into account the literature databases to be used, language considerations, key concepts for retrieving the literature items, and construction of the search query. Additionally, here we show the designed query we tentatively implemented on Web of Science.

#### Literature Databases

The literature search should be performed by querying the literature engines PubMed and Web of Science Core Collection. These literature search engines cover an extended range of medical and health informatics–related studies, and the latter additionally includes the fields of biomedical sciences and engineering, which are of high relevance for retrieving relevant data anonymization of related papers. Similar review projects considered the Web of Science Core Collection database as well [34,35].

#### Article Language Considerations

We will include articles published in English for facilitating the selection and screening of identified literature items.

#### Key Concepts and Search Terms

To efficiently find suitable articles, we have proposed 3 categories (concepts) of search terms, reflecting each of the relevant investigation domains of the study objective. The proposed set of search terms can be extended and documented, if necessary, during the literature extraction process.

While the first category (A) relates to data anonymization methods, the second one (B) captures the field of medical CDMs and data standards, and the last one (C) covers the domain of data quality and utility assessment. Table 1 provides an overview of the key concepts and the explicit search terms.

**Table 1:** Key concepts.

Key concepts	Search terms	Investigation domains
A	Deidentification/ De-identification k-anonymity t-closeness l-diversity Differential privacy De-identified Data masking Data generalization Data perturbation Data permutation Data suppression Data anatomization	Formal data anonymization
B	i2b2 TranSMART OMOP OHDSI CDISC ODM PCORnet FHIR	Medical research CDMs^a^ or data standard
C	Data quality Data accuracy Data utility Data fidelity Fitness for use Fitness for purpose	Assessment of quality or utility of data

^a^CDM: common data model.

#### Search Query Construction

Based on the defined key concepts and search terms, we built a search string by combining the domain of formal data anonymization with those of CDM standards and data quality by using corresponding “AND” and “OR” Boolean operators.

The final search string is built using the following key concept combination:

Search query = A AND (B OR C)

The proposed citation search query is documented in Multimedia Appendix 1.

### Step 3—Study Selection

After the collection of articles meeting the eligibility criteria, a diligent selection process will be followed. This will be based on independent reviews by 2 experts, while a third expert will ensure that a compromise is achieved in case of selection conflicts. Two major stages will constitute this paper selection process.

First, a general screening review based on the title and abstracts of each article will be carried out in order to exclude all references not useful to achieve the targeted research objective.

In the second phase, a content review will be conducted via a full-text reading of each remaining citation included, to determine their final eligibility by considering their relevance for responding to the research questions. In addition, we will document and provide a list of all excluded articles in a complementary appendix.

These 2 phases will be implemented independently by the 2 citation reviewers by using the free web-based application Rayyan [36]. This application supports the traceable management of the inputs of the different contributing stakeholders and transparent conflict management [36]. Thus, any conflict regarding the final decision about the inclusion or exclusion of a reference will be discussed and decided under consideration of the both reviewers’ viewpoints and input from the independent expert; this will be followed by interactive literature explorations within the Rayyan platform in a nonblinded form. Finally, a detailed description of the literature selection process and conflict management will be provided using a PRISMA-ScR (Preferred Reporting Items for Systematic Reviews and Meta-Analyses Extension for Scoping Reviews) flowchart [33].

### Step 4—Extracting and Charting the Data

We will extract from each of the selected articles all relevant information (including metadata) and record these into a template-based documentation, so that a subsequent descriptive analysis (including information visualization) can be performed by using an appropriate statistics package. A general template has been provisionally proposed (see Table 2) considering approaches from similar review projects [34]. Updates on this template will be iteratively and collaboratively integrated, in accordance with requirements during the review, taking into account the concrete relevance for responding to the research objectives.

**Table 2:** Template to extract key information form the included articles.

Metadata	Description
Citation details	Name of first author and coauthors, digital object ID, and journal name
Year of publication	Year of publication of the article in a valid year format (eg, YYYY)
Study type	Use case, framework development, evaluation, etc
Study location	Continent, country, or city hosting the study
Institute	Research institution of the first author
Funding source	Public, industry, or missing
Aims of the study	Objective of the study
Methodology (including technical implementation)	Methods, techniques, models, framework, or approach implemented to achieve the research aims
Study populations (if described in the article)	Targeted research cohort, built on the basis of corresponding eligibility criteria
Summary of outcome measures	Summarizing the study results
Limitations or gaps	Strength and limitations of the study
Important results associated with research question 1	Description of formal relational data anonymization processes on CDM^a^-converted health care data
Important results associated with research question 2	Description of existing evidence to address anonymous data quality and utility: description of implemented strategies and description of observable gaps
Important results associated with research question 3	Description of differences in CDMs regarding how data anonymization modifies the specified table’s granularity and how anonymized data are there recorded

^a^CDM: common data model.

### Step 5—Collating, Summarizing, and Reporting the Results

We will carry out a narrative quantitative analysis of findings using a 2-way analytical framework [33], which will include a descriptive and thematic-based approach. This will generate comprehensive results, outlining the current evidence and research gaps related to the research questions. In doing so, we will first describe the implementation of data anonymization on FHIR- and CDM-formatted data, which include i2b2 TranSMART, OMOP OHDSI, PCORnet, and the CDISC. This will be accompanied by an analysis of deployment to ensure strategies for quality and utility assessment of anonymous data obtained, to present the current state of the art, and identify open research aspects. In addition, the effects of data anonymization on CDM specifications will be presented and discussed.

Furthermore, corresponding comparison tables and graphs (PRISMA-ScR model–oriented) will be presented. Second, the findings will be organized, analyzed, and discussed in accordance with the 2 research questions. A thematically oriented illustration will be additionally generated.

## Results

Following the methodological elements, outlined in steps 2 (identifying the relevant studies) and 3 (study selection), we were able to generate a set of search keywords and design an appropriate literature search query. Furthermore, a tentative execution of this query on Web of Science resulted in the detection of 507 matching publications. In alignment with the presented methodology, these articles will be interactively scrutinized by the experts in order to gain relevant information regarding the research questions. This preparatory work will support the transparent execution of this scoping review study. In doing so, we intend to implement the full extraction of the literature and to proceed with the full execution of the review study by the end of the fourth quarter of 2023.

## Discussion

During the planning stage, we designed and implemented a query allowing the identification of potentially eligible publications, in order to investigate the current status of evidence regarding data quality–preserving relational anonymization of CDM-converted health care data. The considerable amount of eligible literature obtained from Web of Science showed that useful information could be found to describe how relational data anonymizations are performed in the context of CDM-transformed health data and to what extent the quality and utility of obtained anonymous data are addressed in consideration of CDM specifications.

However, a more detailed analysis of these citations should support (1) investigating how the several privacy models, data transformation techniques, and utility models [1] are applied on CDM-converted health data, and (2) document the findings into the CDM databases. Moreover, the obtained set of literature could cover a wide range of current formal anonymization techniques, technologies related to Extraction-Transformation-Load processes for converting source data to the CDM format, or numerous data quality assessment frameworks. This requires a meticulous literature analysis strategy to include the most pertinent citations, which should enable answering the research questions. By following up on the systematic review of Fernández-Alemán et al [37], revealing the necessity of complementary work concerning the security and privacy of EHR data systems, and the investigation by Majeed and Lee [1], presenting the quantification of both utility and privacy of anonymized sensitive data for some scenarios as a challenging task, this scoping review should serve as a response to these questions, capture and describe the current evidence about utility-preserving anonymization of tabular CDM-based health data, and help identify potentially existing research gaps. This aspect is adequately in line with some of the main goals for conducting a scoping review as proposed by Arksey and O’Malley [33], which are to summarize and disseminate research findings and to identify research gaps in the existing literature.

Nevertheless, the planned scoping review might include some restrictions. Regarding the scope of the intended literature review, just a focus on formal data privacy models should be addressed, including, for instance, the k-anonymization, *l*-diversity, differential privacy, and *t*-closeness privacy models. Moreover, only the relational (table-based) data anonymization methods should be approached due to their frequent application for anonymizing tabular data in the medical context. A follow-up review including further anonymization frameworks such as social network– or graph-based data anonymization [1] in the clinical context could be subsequently initiated. However, to address the four-eyes principle on the proposed literature search string early, we will proceed with the validation of the search query by a librarian from the licensed library of Medical Faculty Mannheim, Heidelberg University, in order to correspondingly mitigate any potential conceptual or technical issues in the query.

Among other aspects, it is pertinent to point out that the anticipated definition of the study’s specifications is an essential approach for limiting decision conflicts and providing transparency in the completion of this literature review. This should foster a reproducible and transferable methodology and disseminate reliable insights necessary to enhance and to better understand the approaches for preserving patient privacy and data quality in the secondary use of harmonized health care data.

## Appendix

Multimedia Appendix 1 Revised literature search query.

## Abbreviations

CDISC: Clinical Data Interchange Standards Consortium
CDM: common data model
EHR: electronic health record
FHIR: Fast Healthcare Interoperability Resources
i2b2: Informatics for Integrating Biology & the Bedside
MIRACUM: Medical Informatics in Research and Care in University Medicine
ODM: Operational Data Model
OHDSI: Observational Health Data Sciences and Informatics
OMOP: Observational Medical Outcomes Partnership
PCORnet: Patient-Centered Outcomes Research network
PRISMA-ScR: Preferred Reporting Items for Systematic Reviews and Meta-Analyses Extension for Scoping Reviews

## Data Availability

Scripts for the technical implementation of citation visualization based on statistical software such as RStudio [38], as well as files containing the collected literature and other study documents, will be made available on an open publicly available repository such as Zenodo [39]. These data will be available in an anonymized format for facilitating more transparency and for offering the possibility to reproduce the literature extraction, charting, and analysis processes.
